# Wide Surgical Margin Improves the Outcome for Patients with Gastrointestinal Stromal Tumors (GISTs)

**DOI:** 10.1007/s00268-018-4498-9

**Published:** 2018-02-12

**Authors:** Jan Åhlén, Fredrik Karlsson, Johan Wejde, Inga-Lena Nilsson, Catharina Larsson, Robert Bränström

**Affiliations:** 1 Endocrine and Sarcoma Surgery Unit, Department of Molecular Medicine and Surgery, Karolinska Institutet, Karolinska University Hospital, P9:03, 171 76 Stockholm, Sweden; 2 Department of Oncology-Pathology, Cancer Center Karolinska (CCK), Karolinska Institutet, Karolinska University Hospital, Stockholm, Sweden

## Abstract

**Background:**

Surgical resection is still the main treatment for gastrointestinal stromal tumor (GIST), and R0 excision, regardless of surgical margins, is considered sufficient.

**Methods:**

A cohort of 79 consecutive GIST cases treated at the Karolinska University Hospital, who were without metastasis at diagnosis and who had not received any pre-or postoperative treatment with tyrosine kinase inhibitors, was included. Surgical margins were evaluated at the time of surgery and classified as wide, marginal or intralesional. Time to local/peritoneal recurrence, distant metastasis, and survival were recorded. Cox regression analysis was used to investigate the association between surgical margin, and recurrence and survival.

**Results:**

Local/peritoneal recurrence was diagnosed in 2/39 cases with wide margins, in 7/22 cases with marginal margins, and in 13/18 cases with intralesional surgery. Compared to wide margins this gives a hazard ratio of 6.8 (confidence interval 1.4–32.7) for marginal margins and 13.5 (3–61) for intralesional margins. In multivariate analysis, adjusting for size, site, and mitotic index, surgical margin remained an independent significant predictor of risk for recurrence. When classifying patients according to R0/R1 surgery, patients with R0 surgery showed longer time to peritoneal recurrence and better recurrence-free and disease-specific survival as compared to those with R1 resection. However, when excluding patients operated with wide surgical margin, no significant difference was observed.

**Conclusion:**

Wide surgical margins are of significant prognostic importance, supporting the strategy of en bloc resection with good margin and careful handling of the tumor to avoid damaging the peritoneal surface in surgical resection of GIST.

**Electronic supplementary material:**

The online version of this article (10.1007/s00268-018-4498-9) contains supplementary material, which is available to authorized users.

## Introduction

Gastrointestinal stromal tumor (GIST) is the most common mesenchymal tumor in the gastrointestinal tract [1]. The annual incidence is 1.1–1.5 per 100,000 habitants [2, 3] and the prevalence is increasing [4], mainly due to the introduction of tyrosine kinase inhibitors (TKI) that has substantially prolonged patient survival [4]. However, surgery is the main treatment, both for patients with localized disease and patients with locally advanced disease and neo-adjuvant TKI treatment. In population-based cohorts of GIST-patients treated with surgery alone, the 15-years recurrence-free survival was estimated to 60% [5]. In addition, large tumor size, high mitotic count, non-gastric location, presence of tumor rupture and male gender are negative prognostic factors [5].

A surgical R0 resection (i.e. microscopically negative margin) is regarded as a prerequisite for curative intended treatment [6], while tumor rupture is associated with a worse prognosis [5, 7]. However, the general opinion is that R0 resection is sufficient regardless of surgical margins, and R1 resection (i.e. microscopically positive margin) has in some studies shown similar prognosis as R0 resection [8, 9]. In other soft tissue sarcomas, surgery is classified based on resection margins into intralesional, marginal or wide [10]. This subgrouping of R0 and R1 surgery has prognostic significance, and resection margins of 2–3 cm provide good local control [11, 12]. Although this classification has not been proven to be applicable for GIST, an extensive surgical technique gave very good results [13]. In addition, no recurrence was observed during follow-up of 31 patients operated with mainly laparoscopic technique, emphasizing the value of careful resection of all tumors with undamaged peritoneal covering and at least 2 cm margins in site of origin and adherent tissue [14].

Here we determined the prognostic value of surgical resection margins in GIST patients.

## Patients and methods

### Patients

In this observational study, we have retrospectively analyzed the outcome of consecutive GIST patients admitted to the Karolinska University Hospital in Stockholm, Sweden during the period June 1981 to January 2010. The entire cohort included 129 patients where surgical margin was assessed prospectively at the time of surgery. 79 patients without metastasis at diagnosis and no TKI treatment (GIST-nonMET/nonTKI), were included in the main analyses after exclusion of 14 patients with metastatic disease at diagnosis and 36 patients without metastases at diagnosis but who had received (neo)adjuvant TKI treatment (Table 1). Analyses were also carried out for all 115 patients who were without metastasis at diagnosis regardless of TKI treatment (GIST-nonMet).

**Table 1:** Clinical details and follow-up for the 79 GIST cases in the GIST-nonMET/nonTKI cohort

Parameter (*n* = informative cases)	GIST-nonMET/nonTKI		Wide margin		Marginal margin		Intralesional margin	
	Number (%)	Time/rec	Number (%)	Time/rec	Number (%)	Time/rec	Number (%)	Time/rec
Age at diagnosis (*n* = 79)	(*n* = 79)		(*n* = 39)		(*n* = 22)		(*n* = 18)	
Median (min–max) year	–	61 (10–86)	–	67 (10–86)	–	58 (36–85)	–	56 (19–82)
Gender (*n* = 79)								
Male	34 (43%)	–	14 (36%)		13 (59%)		7 (39%)	
Female	45 (57%)	–	25 (64%)		9 (41%)		11 (61%)	
Tumor localization (*n* = 79)								
Esophagus	2 (2%)	–	–	–	1 (4%)	0	1 (6%)	1
Ventricle	45 (57%)	–	28 (72%)	2	12 (54%)	5	5 (28%)	3
Duodenum	5 (6%)	–	–	–	2 (9%)	1	3 (17%)	3
Small intestine	21 (27%)	–	10 (25%)	2	5 (23%)	4	6 (33%)	5
Colon	3 (4%)	–	–	–	1 (4%)	1	2 (11%)	2
Rectum	3 (4%)	–	1 (3%)	0	1 (4%)	1	1 (6%)	1
Tumor size (*n* = 78)								
>10 cm	17 (21%)	–	4 (10%)	1	5 (23%)	4	8 (47%)	8
>5–10 cm	19 (24%)	–	10 (26%)	2	7 (32%)	3	2 (11%)	1
2–5 cm	36 (47%)	–	21 (54%)	1	8 (36%)	4	7 (41%)	5
<2 cm	6 (8%)	–	4 (10%)	0	2 (9%)	1	–	–
Surgical margin (*n* = 79)								
Wide	39 (49%)	–	39	4	–	–	–	–
Marginal	22 (28%)	–	–	–	22	12	–	–
Intralesional	18 (23%)	–	–	–	–	–	18	15
Mitotic index (*n* = 72)								
High ≥ 10	11 (14%)	–	2 (6%)	1	2 (9%)	2	7 (44%)	7
Intermediate 5–9	23 (28%)	–	14 (40%)	2	7 (33%)	5	2 (12%)	1
Low < 5	38 (48%)	–	19 (54%)	1	12 (57%)	5	7 (44%)	5
Risk (*n* = 79) (Joensuu [15])								
High risk	28 (37%)	–	6 (15%)	1	9 (41%)	7	13 (72%)	13
Intermediate risk	15 (20%)	–	11 (28%)	3	4 (18%)	2	–	–
Low or very low risk	36 (43%)	–	22 (56%)	0	9 (41%)	3	5 (28%)	2

Patients were characterized for demographic variables, tumor characteristics, risk criteria and treatment and were followed up concerning local/peritoneal recurrence, distant metastasis and survival. Risk criteria used for recurrence were size, site and mitotic index according to Joensuu [15]. Tumor size was estimated from the histopathological reports. Based on the largest diameter, the tumors were subgrouped as <2 cm, ≥2 to <5 cm, ≥5 to ≤10 cm or >10 cm. Mitotic index was defined as the number of mitosis observed in 50 high-power fields. The site of the primary tumor was described according to the location as well as grouped as gastric (ventricle) or non-gastric (esophagus, duodenum, small intestine, colon, or rectum). Local/peritoneal recurrence was defined as recurrence at the site of origin or peritoneal recurrence. Metastatic disease was defined as recurrence at a distant location, most commonly the liver.

### Classification of surgical margin

Surgical margins were assessed at the time of surgery according to definitions below. If the distance was doubtful, measurement stick was used. The result was supplemented by histopathological examination and documented as wide, marginal or intralesional. Classification of R0, R1 and R2 surgery were according to Wittekind et al. [16]. The criteria used for definitions of surgical margins were those used for sarcomas in other locations originally defined by Enneking et al. [10], adapted to the abdominal cavity [12].

**Fig. 1:**
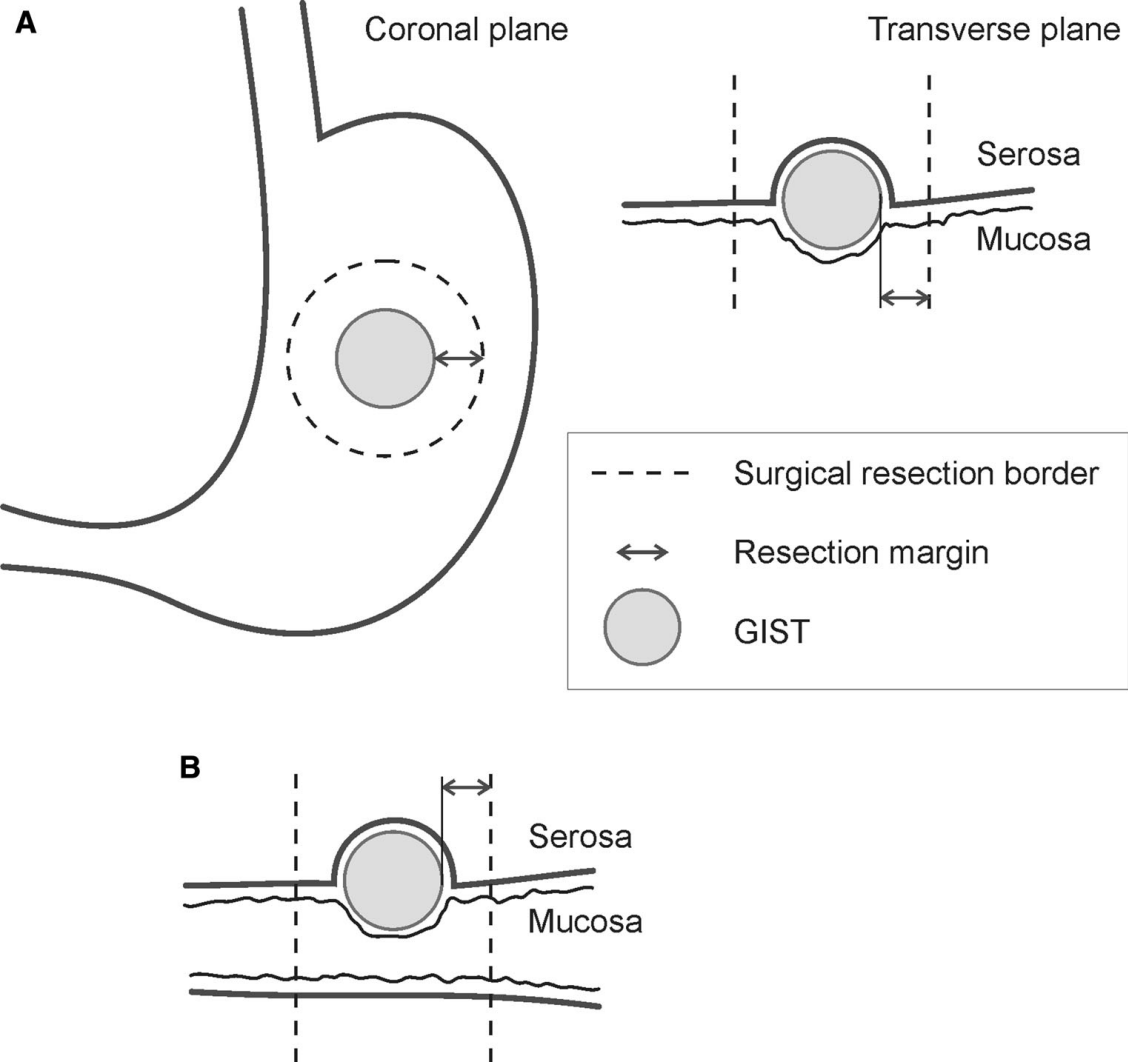
Schematic drawing of surgical margins in gastric (**a**) and non-gastric (**b**) GIST resection. The dotted line indicates the plane of resection, and arrows denote surgical margin. The resection is considered wide when the smallest margin exceeds, or equals, 2 cm and intact serosa covers the tumor. A marginal margin is defined as resection with less than 2 cm, and intact serosa, whereas intralesional margin is defined as the presence of tumor cells in the resection border and/or non-intact covering serosa, peritoneum or adventitia. For more details in the definition of surgical margins, see section “Patients and methods, Classification of surgical margin”

- Wide margin was defined as resection with at least 2 cm normal tissue from the lesion to resection line in the organ of origin, and intact adventitia, serosa, or peritoneum covering the tumor in other directions (Fig. 1). In the case of tumor overgrowth, en bloc resection with at least 2 cm of normal tissue surrounding the tumor was also considered as wide margin.
- Marginal margin meets the same criteria as wide margin, except that the distance from the lesion to resection line is less than 2 cm. This includes an intact adventitia, serosa, or peritoneum covering the tumor in other directions. Notably, both wide and marginal margin are R0 resections.
- Intralesional margin was defined as R1 resection (microscopic or macroscopic tumor residuals at the resection border), which includes incision into the tumor, tumor rupture, enucleation or absent/damaged covering adventitia, serosa, or peritoneum.

Since patients with macroscopic residual tumor were classified as having persistent disease and always treated with TKI, R2 cases were excluded.

### Statistics

Statistical analyses were performed using StatSoft, Inc. (2011) Statistica version 12 (www.statsoft.com). Because data did not follow a normal distribution, they are expressed as median and range. The Kaplan–Meier method and log-rank test were used to compare time from diagnosis to local/peritoneal recurrence and metastasis [recurrence-free survival (RFS)], time to death from any cause [overall survival (OS)] and time to death from disease [disease-specific survival (DSS)] as described by National Cancer Institute (NCI). DSS does not account for patients who die from other causes unrelated to GIST.

Prognostic factors were compared by multivariate analysis using Cox proportional hazards model. All tests were done two‐tailed, and *P* values <0.05 were considered to be statistically significant.

## Results

### Improved outcome from wide surgical margins in GIST patients

Clinical details and follow-up for GIST-nonMet/nonTKI are summarized in Table 1, survival data specifying wide, marginal and intralesional margin in Table 2, and number of events according to margin and size in Supplementary Table S1. The median follow-up for patients with non-recurrent disease was 76 months (range 10–179 months). Size of the primary tumor (*P* = 0.01), mitotic index (*P* = 0.003), tumor site as gastric or non-gastric (*P* = 0.003), but not gender were associated to risk of any first event. Risk of death from disease was associated to size (*P* = 0.004), mitotic index (*P* = 0.03), tumor site as gastric or non-gastric (*P* = 0.05), but not to gender. The 5-year RFS in this group was 76%. During follow-up, 31/79 patients (39%) were diagnosed with local/peritoneal recurrence and/or distant metastases, mainly to the liver (Table 1). Among these 31 patients, 22 (28%) exhibited local/peritoneal recurrence, 17 (22%) distant metastases and thus 8 patients had both local/peritoneal recurrence and distant metastasis. Thirteen patients (16%) died from disease during follow-up, of which 5 died from local/peritoneal disease without distant metastasis and for the entire observation period the overall survival was 66% (Table 2). The 5-year OS was 86% (11 of 79 patients died). In the wide group 2/39 patients died, none from disease. In the marginal group 5/22 died, two from disease. Median time to death was 32 months (range 13–51). In the intralesional group, 4/18 patients died within 5 years, all of whom died from disease at a median follow-up of 18.5 months (range 4–51). The median time to any event, local/peritoneal recurrence or metastasis, was 45 months (mean 60, SD 58, range 1–274 months), to local/peritoneal recurrence 48 months (mean 65, SD 66, range 1–274) and to metastasis 44 months (mean 68, SD 83, range 6–361). Median time to death from disease was 86 months (mean 95, SD 65, range 4–388 months).

**Table 2:** Survival of patients with wide, marginal and intralesional margin in the GIST-nonMET/nonTKI cohort

Follow-up survival (observational time)	All cases (*n* = 79)		Wide (*n* = 39)		Marginal (*n* = 22)		Intralesional (*n* = 18)	
	Survival (events)	Time to event median (min–max) months	Survival (events)	Time to event median (min–max) months	Survival (events)	Time to event median (min–max) months	Survival (events)	Time to event median (min–max) months
Recurrence-free survival	61% (31)	45 (2–274)	90% (4)	79 (45–127)	45% (12)	44 (5–122)	17% (15)	32 (2–274)
Disease-specific survival	84% (13)	72 (4–194)	100% (0)	–	77% (5)	79 (13–167)	56% (8)	61 (4–194)
Overall survival	66% (27)	71 (4–194)	80% (8)	69 (10–110)	50% (11)	73 (13–167)	56% (8)	61 (4–194)

**Fig. 2:**
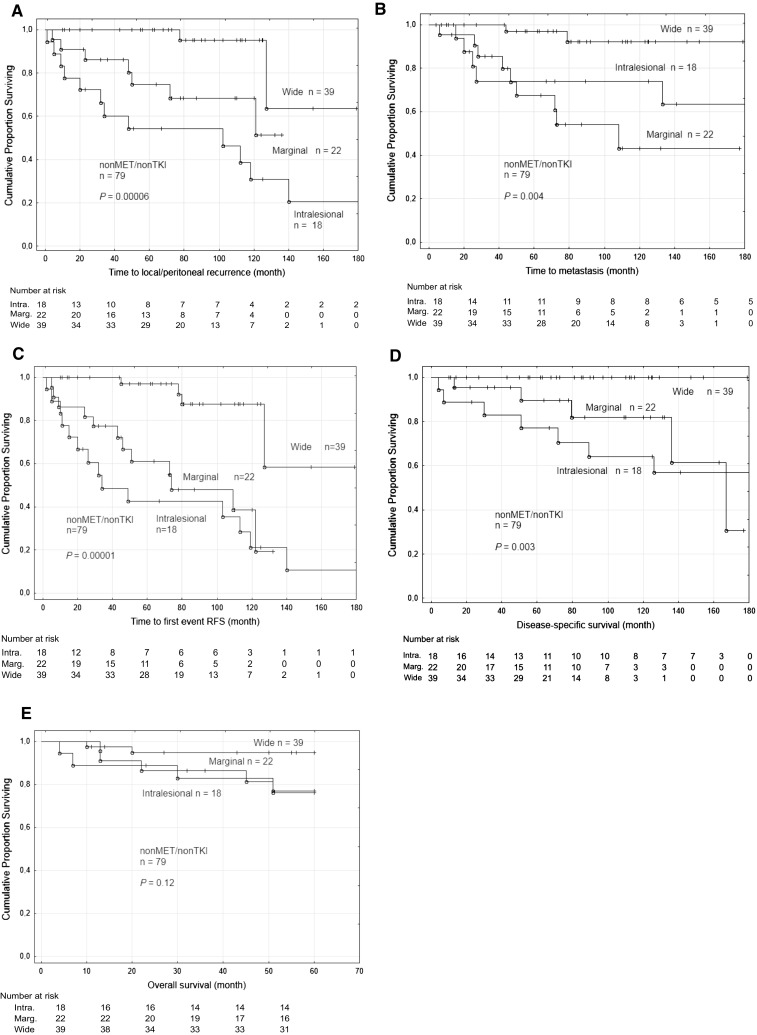
Outcome according to surgical margin among the 79 patients in the GIST-nonMet/nonTKI group. Kaplan-Meier curves showing time to local/peritoneal recurrence (**a**), time to metastasis (**b**), recurrence-free survival (**c**), disease-specific survival (**d**), and overall survival (**e**) for patients with wide, marginal or intralesional surgical margins

Analyses of surgical margins in GIST-nonMet/nonTKI revealed a higher risk for local/peritoneal recurrence for patients with marginal margin HR 6.8 (1.4–32.7) and intralesional margin HR 13.5 (3–61) as compared to wide margin (*P* = 0.003) also shown in Fig. 2a using the Kaplan-Meier survival estimates (*P* = 0.00006, Table 3). Wide margin was associated to longer time to metastasis (*P* = 0.004, Fig. 2b) longer RFS (*P* = 0.00001, Fig. 2c) and DSS (*P* = 0.003, Fig. 2d) but not OS (Fig. 2e).

**Table 3:** Multivariate analyses of hazard ratios (HR) for local/peritoneal recurrence according to surgical margin

Variable	Compared to wide margin	GIST-nonMet/nonTKI (*n* = 79)		GIST-nonMet (*n* = 115)	
		Hazard ratio (limits)	*P* value*	Hazard ratio (limits)	*P* value*
None	Marginal	6.8 (1.4–32.7)	0.003	5.3 (1.1–25.1)	0.003
	Intralesional	13.5 (3–61)		11.7 (2.6–51.5)	
Size (diameter)	Marginal	7.0 (1.4–34.1)	0.01	5.6 (1.2–27.3)	0.01
	Intralesional	11.1 (2.2–55.2)		10.2 (2.1–48.7)	
Site (gastric/non-gastric)	Marginal	6.5 (1.3–31.2)	0.007	5.1 (1.1–24)	0.006
	Intralesional	11.5 (2.5–53.8)		10.4 (2.3–46.8)	
Mitotic index	Marginal	7.9 (1.6–38.2)	0.02	5.0 (1.1–23.7)	0.059
	Intralesional	7.6 (1.5–37.6)		6.5 (1.4–30.9)	
Combining all above (size + site + mitotic index)	Marginal	7.4 (1.4–38.3)	0.048	5.6 (1.1–28.1)	0.081
	Intralesional	6.0 (1.1–32.7)		6.2 (1.1–33.4)	

*P value calculated according to Wald test

The surgical margin remained a significant predictor of risk of recurrence also when the gastric (*P* = 0.025) and non-gastric GISTs (*P* = 0.023) were analyzed separately (Supplementary Fig. S1).

In multivariate analysis with the parameters surgical margin, tumor size, mitotic index, and site, only surgical margin (*P* = 0.02) and mitotic index (*P* = 0.04) were identified as independent parameters associated to local/peritoneal recurrence. Surgical margin remained an independent risk factor of local/peritoneal recurrence after adjusting for tumor size, mitotic index, and site (Table 3). When including tumor size in the analysis marginal margin still had a HR of 7.0 (1.4–34.1) and intralesional margin a HR of 11.1 (2.2–55.2) compared to wide margin (*P* = 0.01, Table 4). When including site as gastric or non-gastric, marginal margin resulted in a HR of 6.5 (1.3–31.2) and intralesional margin in a HR of 11.5 (2.5–53.8) (*P* = 0.007, Table 3). Adding mitotic index to marginal margin results in HR 7.9 (1.6–38.2) and for intralesional margin 7.6 (1.5–37.6) (*P* = 0.02, Table 3).

**Table 4:** Events at follow-up according to surgical margin and size of tumor

Surgical margin tumor size	Patients (*n* =)	Number of cases with			
		Recurrence any type	Recurrence local/peritoneal	Distant metastasis	Metastasis + local/peritoneal recurrence
Intralesional					
≥10 cm	8	8	6	5	3
≥5 < 10 cm	2	1	1	1	1
≥2 < 5 cm	7	5	5	0	0
<2 cm	0	0	0	0	0
All	17	14	12	6	4
Marginal					
≥10 cm	5	4	3	3	2
≥5 < 10 cm	7	3	2	1	0
≥2 < 5 cm	8	4	1	4	1
<2 cm	2	1	1	1	1
All	22	12	7	9	4
Wide					
≥10 cm	4	1	1	0	0
≥5 < 10 cm	10	2	1	1	0
≥2 < 5 cm	21	1	0	1	0
<2 cm	4	0	0	0	0
All	39	4	2	2	0
Total	78	30	21	17	8

### Analysis of outcome using R0/R1 classification

**Fig. 3:**
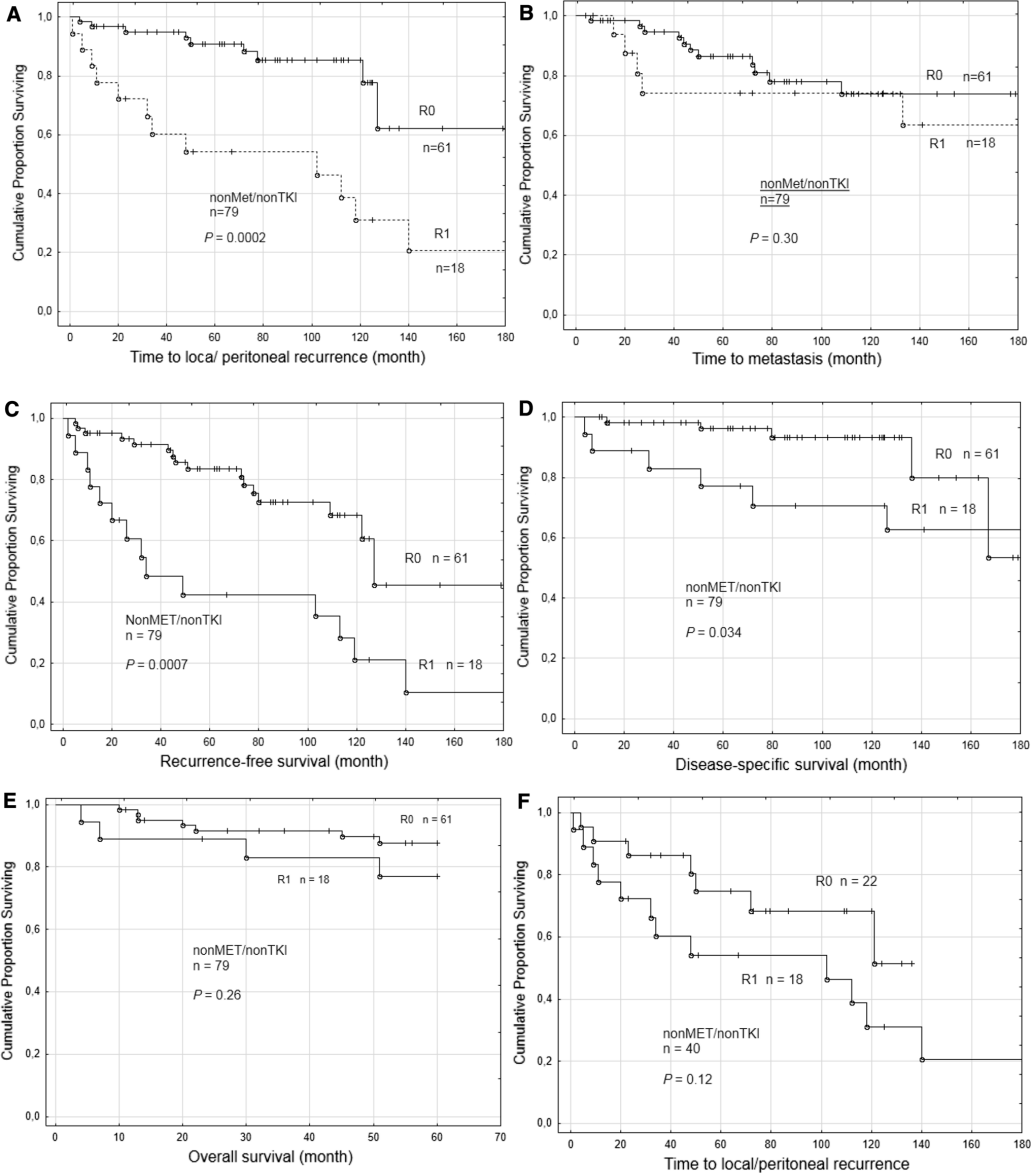
Outcome according to R0 or R1 surgery in the GIST-nonMet/nonTKI group. Kaplan-Meier curves illustrate time to local/peritoneal recurrence (**a**), time to metastasis (**b**), recurrence-free survival (**c**), disease-specific survival (**d**), and overall survival (**e**) for the 79 patients in the GIST-nonMet/nonTKI group patents with R0 (microscopically negative margin) or R1 (microscopically positive margin) surgical resection according to [16]. Comparisons of time to local/peritoneal recurrence in the subset of patients with marginal margin as R0 with intralesional margin R1 (**f**). Patients with R2 surgery (macroscopic residual tumor) were always TKI treated and therefore not included

The outcome was also evaluated in relation to residual tumor classification [16] to compare R0 resection (wide and marginal margins, both defined as R0 resection) with R1 resection (intralesional) in GIST-nonMet/nonTKI. R1 resection had a significantly higher risk for local/peritoneal recurrence (*P* = 0.0002, Fig. 3a) and was associated to shorter RFS (*P* = 0.0007, Fig. 3c), and DSS (*P* = 0.034, Fig. 3d), but not to time to metastasis (Fig. 3b) or OS (Fig. 3e). Furthermore, when patients operated with wide margins were excluded from the analysis, thus comparing only marginal margin (R0) to intralesional margin (R1), there was no significant difference in risk for local/peritoneal recurrence (*P* = 0.12, Fig. 3f).

### Improved outcome for wide surgical margin in patients without metastasis at diagnosis but including TKI treated patients

Among the 115 patients in GIST-nonMet, wide margin was associated to longer time to local/peritoneal recurrence and metastases and longer RFS, OS, and DSS (Supplementary Fig. S2). Compared to wide margin, marginal margin gave a HR of 5.3 (1.1–25.1) and intralesional margin a HR of 11.7 (2.6–51.5, *P* = 0.003) for local/peritoneal recurrence. In multivariable analysis, including size and site, wide margin was still independently associated to lower risk for local/peritoneal recurrence (Table 3). When including size, marginal margin had a HR of 5.6 (1.2–27.3) and intralesional margin a HR of 10.2 (2.1–48.7) (*P* = 0.01). Similarly, when site was included marginal margin had a HR of 5.1 (1.1–24) and intralesional margin a HR of 10.4 (2.3–46.8) (*P* = 0.006) (Table 3).

## Discussion

The major finding in this study is the prognostic importance of surgical margin in GIST. The classical system for surgical staging of musculoskeletal sarcomas [10] stresses the importance of surgical margins consisting of uninvolved anatomical barriers. The definition is designed for lesions in the extremities and includes longitudinal and vertical margins. This concept is not directly applicable for tumors expanding into peritoneal cavity like GISTs [10]. Therefore, in this study, wide surgical margin was defined as the smallest distance of 2 cm from the lesion to resection line in the organ of origin, and an intact adventitia, serosa, or peritoneum covering the tumor in other directions (Fig. 1). In the case of tumor overgrowth, en bloc resection with at least 2 cm of normal tissue surrounding the tumor was also considered as wide margin. Marginal margin, meets the same criteria as wide margin, except that the distance from the lesion to resection line is less than 2 cm, but still R0 resected. Intralesional margin was defined as resection with micro-or macroscopic tumor residuals at the resection border, including incision into the tumor, tumor rupture, and enucleation or absent/damaged covering serosa, or peritoneum.

Wide surgical margin was associated with a reduced risk of local/peritoneal recurrence, metastatic disease and longer DSS as compared to marginal and intralesional margins. 5-year OS showed a non-significant tendency for longer survival (*P* = 0.12, Fig. 2e). However, there were only 11 events among the 79 patients and only 2/39 died within 5 years in the wide group, whereas 5/22 in the marginal group and 4/18 in the intralesional group. Furthermore, none in the wide group died from disease while 2 in the marginal and all 4 in the intralesional group died from disease.

To our knowledge, this is the first study that shows the importance of the extent of surgical excision in GIST. Wide surgical margins reduced the risk of recurrence and prolonged RFS and DSS compared to intralesional margin and, importantly, compared to marginal margin. In multivariate analyses, these findings remained independent of tumor size, site, and mitotic index. The observations are in agreement with other types of soft tissue sarcoma, where it is well established that patients operated with wide margins have a better outcome [11, 12, 17]. Interestingly no patient with wide margin has so far died from the disease. Furthermore, events were only observed in 4/39 patients with wide compared to 12/22 with marginal and 15/18 with intralesional margin.

When including patients that were treated with TKI in addition to surgery, essentially similar results were obtained with a significant association between wide margin and lower risk of local/peritoneal, metastatic disease and death from the disease. Although based on a limited number of samples, the difference when including these patients is somewhat lower which might indicate that TKI has a higher impact on recurrence in the marginal and intralesional group.

The observed association between intralesional margin and worse prognosis was not surprising. Ruptured GISTs are known to have a poor prognosis [5, 7, 18] and R1 resection must be regarded as almost equal to a ruptured tumor although no gross tumor is left behind. Our data are also in agreement with several other studies reporting that patients treated with R1 resection do worse than those with R0 resection [7, 19, 20]. As expected, when combining wide and marginal margins (both R0), the risk of local/peritoneal recurrence as well as the DSS were significantly better than for intralesional margin (R1), whereas there was no longer any significant difference for distant metastasis. Importantly, we did not observe any significant difference in outcome between R0 and R1 when excluding patients with wide surgical margin, thus comparing marginal margin (R0) with intralesional margins (R1). This supports the assumption that the difference between R0 and R1 is related mainly to wide margins, although we must consider the risk of type II error.

In conclusion, our findings imply that GIST should be treated according to surgical strategies used for other types of sarcomas. We show that wide surgical margin is an important prognostic factor for GIST patients supporting the strategy of en bloc resection with good margins. In our experience this rarely causes any disability.

## Electronic supplementary material

Below is the link to the electronic supplementary material.

Supplementary material 1 (PDF 93 kb)

Supplementary material 2 (PDF 125 kb)

Supplementary material 3 (PDF 53 kb)
