# Supplementary material for: Wide Surgical Margin Improves the Outcome for Patients with Gastrointestinal Stromal Tumors (GISTs)
Source: World J Surg. 2018 Feb 12;42(8):2512–21. doi: 10.1007/s00268-018-4498-9 (PMC6060789; doi:10.1007/s00268-018-4498-9)
Supplement: Supplementary file 1 — Supplementary material 1 (PDF 93 kb) [file 268_2018_4498_MOESM1_ESM.pdf]

Supplementary Fig. S1

A

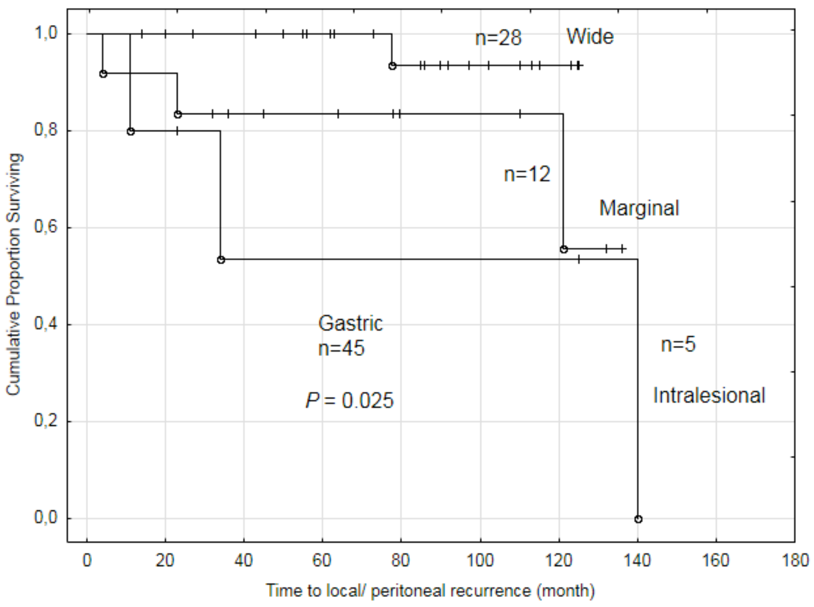

B

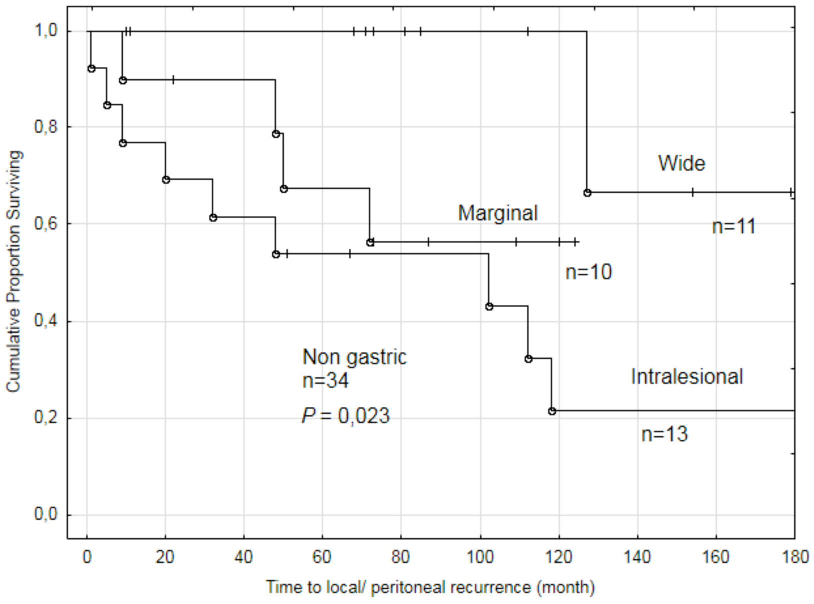

Figure S1. Outcome according to surgical margin for gastric and non-gastric tumor location among the 79 patients in the GIST-nonMet/nonTKI group. Kaplan-Meier curves show time to local/peritoneal recurrence in patients with gastric (a) and non-gastric (b) tumor location.
