# Supplementary material for: Wide Surgical Margin Improves the Outcome for Patients with Gastrointestinal Stromal Tumors (GISTs)
Source: World J Surg. 2018 Feb 12;42(8):2512–21. doi: 10.1007/s00268-018-4498-9 (PMC6060789; doi:10.1007/s00268-018-4498-9)
Supplement: Supplementary file 2 — Supplementary material 2 (PDF 125 kb) [file 268_2018_4498_MOESM2_ESM.pdf]

Supplementary Fig. 2

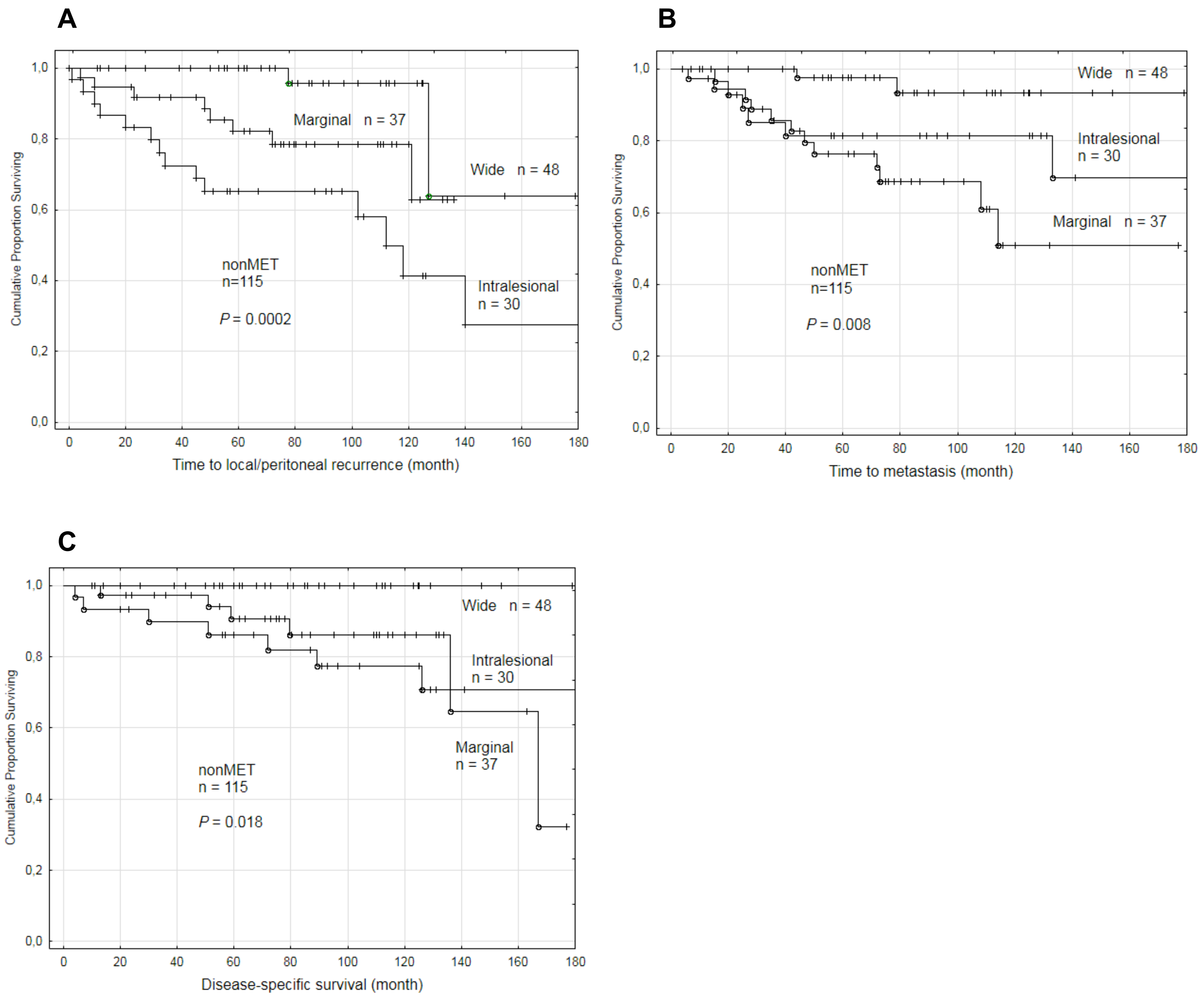

Figure S2. Outcome according to surgical margin among the 115 patients in the GIST-nonMet group. Kaplan-Meier analysis are shown for time to local/peritoneal recurrence (a), time to metastasis (b), and disease-specific survival (c).
