# Supplementary material for: Wide Surgical Margin Improves the Outcome for Patients with Gastrointestinal Stromal Tumors (GISTs)
Source: World J Surg. 2018 Feb 12;42(8):2512–21. doi: 10.1007/s00268-018-4498-9 (PMC6060789; doi:10.1007/s00268-018-4498-9)
Supplement: Supplementary file 3 — Supplementary material 3 (PDF 53 kb) [file 268_2018_4498_MOESM3_ESM.pdf]

Table S1. Events at follow-up according to surgical margin and size of tumor.

Surgical margin<br>Tumor size	Patients<br>(n =)	Number of cases with<br>Recurrence<br>any type	Recurrence<br>Local / Peritoneal	Distant<br>metastasis	Metastasis +<br>Local / Peritoneal Rec
Intralesional					
≥10 cm	8	8	6	5	3
≥5 <10 cm	2	1	1	1	1
≥2 <5 cm	7	5	5	0	0
<2 cm	0	0	0	0	0
All	17	14	12	6	4
Marginal					
≥10 cm	5	4	3	3	2
≥5 <10 cm	7	3	2	1	0
≥2 <5 cm	8	4	1	4	1
<2 cm	2	1	1	1	1
All	22	12	7	9	4
Wide					
≥10 cm	4	1	1	0	0
≥5 <10 cm	10	2	1	1	0
≥2 <5 cm	21	1	0	1	0
<2 cm	4	0	0	0	0
All	39	4	2	2	0
Total	78	30	21	17	8
